# The anti-Alzheimer compounds from tempeh oil in LPS-induced neuronal Schwann cells

**DOI:** 10.1186/s42779-022-00163-2

**Published:** 2022-12-08

**Authors:** Eileen C. Limanjaya, Dionysius Subali, Yanti Yanti

**Affiliations:** Department of Biotechnology, Atma Jaya Catholic University of Indonesia, Jakarta, Indonesia

**Keywords:** Tempeh oil, Omega-3, Anticholinesterase, Antioxidant, Gene expression, Alzheimer’s disease

## Abstract

Tempeh is a traditional fermented Indonesian food from white soybean. Tempe has better nutritional value than non-fermented white soybean. The aim of this study was to extract tempeh oil and analyze the inhibitory potency of Alzheimer-related gene expression in LPS-induced neuronal Schwann cells. Tempeh oil was extracted with Bligh Dyer method and was analyzed with PUFA identification, anticholinesterase activity, antioxidant activity, and quantitative PCR. Tempeh oil had a total yield of 7.14%, and PUFA identification found 8.37% omega-3. The anti-acetylcholinesterase activity showed that tempeh oil 25 µg/mL had the highest activity and 500 µg/mL in anti-butyrylcholinesterase activity. The quantitative PCR showed that tempeh oil had downregulated the gene expression of *PSEN1, Gsk3b, cdk5,* and *TNF*. From this study, tempeh oil may have the potential to lower the risk of Alzheimer’s disease by regulating certain gene traits.

## Background

Alzheimer’s disease is a chronic neurodegenerative disease with dementia as the symptoms which gets worsen over time. The World Health Organization has reported there are 50 million people with dementia and predicted the number will increase up to 82 million people in 2030. In 2016, Indonesia had estimated that 1.2 million people have Alzheimer’s disease and will go up to 4 million people in 2050 [1].

The main cause of Alzheimer’s disease is the accumulation of β-amyloid in the brain that leads to production of protein aggregates, which called plaque, and the aggregates of tau hyperphosphorylation that may interrupt nutrition transport in neuron cells [2, 3]. Altered neurotransmitter activities are often occur in the brain of patients with Alzheimer’s disease, including glutamatergic and cholinergic neurotransmission systems [4, 5]. Acetylcholine is a neurotransmitter utilized by all cholinergic neurons that plays a vital role within the peripheral and central nervous systems [6]. In Alzheimer’s disease, the acetylcholine signaling process is terminated by acetylcholinesterase (AChE), an enzyme that rapidly hydrolyses acetylcholine through several cholinergic pathways that may lead to acetylcholine deficiency [4, 7, 8].

Genetic also plays a role in the development of Alzheimer’s disease. The genetic risk of Alzheimer’s disease is estimated at approximately 70% [9]. Several genes are involved in the etiopathogenesis of Alzheimer’s disease, including *TNF, cdk5, gsk3b, PSEN1,* and *PSEN2.* Neurodegenerative diseases are often linked to chronic inflammation, and TNF-α acts as the main cause of chronic inflammation in the central nervous by producing other pro-inflammatory cytokines [10, 11]. Two kinases, cyclin-dependent kinase 5 (cdk5) and glycogen synthase kinase 3β (GSK3β) have been involved in the regulation of tau hyperphosphorylation, increasing the amyloid β (Aβ) production [12]. Presenilin-1 (PSEN1) and presenilin-2 (PSEN2) genes encode the major component of y-secretase, which is responsible for the formation of amyloid-β peptides [13].

**Fig. 1:**
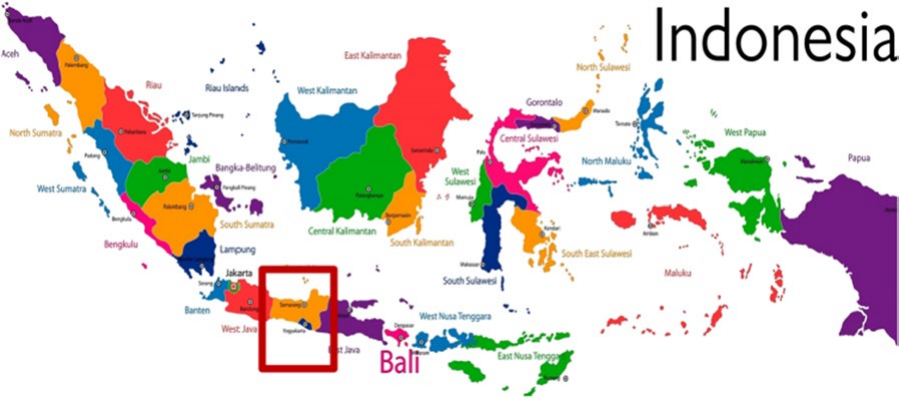
Historical evidence showed that tempeh was originated from Central Java, Indonesia

As a legume native to Eastern Asia [14], soybean is widely consumed in the Eastern and South-eastern Asia countries both as fermented and non-fermented foods. Many Asians believe that incorporating soy-based foods into diets can improve overall well-being and help in preventing chronic diseases [15]. Tempeh is a traditional Indonesian fermented food primarily made from white soybean with *Rhizopus* spp. [16]. Historically, tempeh is believed to have originated in Central Java around the 1700s [17] (Fig. 1). Since then, tempeh has been widely consumed, especially on Java and Bali islands, as a staple protein source due to its high nutritional contents and affordable price [18].

The fermentation process in tempeh making increases its nutritional value [19]. Some biochemical changes occur during the process of soybean fermentation [17], thus improving the bioavailability of protein and polyunsaturated fats while decreasing the anti-nutrients such as phytate, oxalate, trypsin inhibitor, and antinutritive phenols [18]. Tempeh consumption has been reported to provide a wide range of health benefits, mainly due to the presence of bioactive compounds called isoflavones. Isoflavones can potentially act as antioxidants to protect the cells from oxidative stress, thus decreasing the risk of many chronic diseases, such as cardiovascular disease, cancer, and neurodegenerative disease [15]. The functionality of tempeh is also due to the presence of polyunsaturated fatty acids (PUFAs), as soybean is rich in linoleic, oleic, and linolenic acids. PUFAs are involved in the process of fat metabolism and maintain the integrity of cell membranes [20]. A study by Subali et al. [21] demonstrated that tempeh oil mainly consisted of 52.3% linoleic acid but not linolenic acid.

The aim of this study was to extract the tempeh oil and analyze the inhibitory potency of Alzheimer’s-related gene expression in LPS-induced neuronal Schwann cells. Gene expression analysis using quantitative PCR is the most efficient method, if compared to microarray and next-generation sequencing (NGS) to provide a comprehensive data related to the genetic factors of Alzheimer’s disease [22]. Gas chromatography (GC) is usually carried out to identify and analyze fatty acid composition from natural sources [23].

Up to the present time, Alzheimer’s drugs can only decelerate its symptoms and a defined cure has not been found. Cholinesterase (ChE) has emerged as the most relevant therapeutic target for ameliorating symptoms of Alzheimer’s disease [24]. Several drugs such as galantamine, donepezil, and rivastigmine have been used to inhibit the activity of cholinesterase that had function to make the symptoms deteriorate [25]. Since tempeh is widely available throughout the country and is considered very affordable, tempeh can meet the nutritional needs of patients with Alzheimer’s disease to naturally improve their condition and overall well-being, without relying on drugs. This study is expected to promote tempeh, an intangible cultural heritage from Indonesia, as more than just a portion of food, but also as a nutritional source that contributes to the population’s health and overall well-being, by emphasizing the benefits of the nutritional content of tempeh to combat chronic diseases, in this case, Alzheimer’s.

## Methods

### Research method overview

The research was conducted at Laboratory of Biochemistry and Enzyme Technology, Faculty of Biotechnology, Atma Jaya Catholic University of Indonesia from September 2020 to June 2021. There were seven main steps, including tempeh fermentation, tempeh oil extraction, polyunsaturated fatty acid (PUFA) analysis using gas chromatography (GC), enzymatic assay, antioxidant assay, cells treatment, and gene expression analysis using quantitative real-time polymerase chain reaction (qRT-PCR). The materials used for this research were raw soybean (obtained from the Local Market), tempeh starter culture (Raprima Brand), ATCC CRL 2765 (RSC 96) cell culture, acetylcholine iodide (Sigma-Aldrich), acetylcholine esterase from electric eel (Sigma-Aldrich), butyrylcholinesterase from equine serum (Sigma-Aldrich), tripyridyl triazine (TPTZ) (Sigma-Aldrich), sensiFAST cDNA synthesis kit (Bioline), and sensiFAST SYBR LO-ROX kit (Bioline), while the instruments used in this study included microplate reader (TECAN Infinite M2000), inverted microscope (Nikon), and Real-time PCR (Applied Biosystems 7500 Fast).

### Tempeh fermentation

Tempeh was made according to Mubarok et al. [26] with modification. The raw soybean was weighed for 500 grams and rinsed. It was soaked with water for overnight, and then, the skin was removed. The soybean was then boiled for 30 min and cooled until room temperature. The tempeh starter culture was added to the soybean for 1 gram and mixed thoroughly. The soybean was wrapped in banana leaves and incubated in warm temperature about 37 ℃ for 48 h.

### Tempeh preparation and oil extraction

Tempeh was cut into small dices and freeze dried for 2 days. Raw soybean that has been boiled was also freeze dried for control. The extraction process was done using method from Subali et al. [21]. The samples were grounded to powder and extracted using Bligh-dyer method with solvent ratio 1:1:1 for chloroform/methanol/aquadest. The soaked powders were then centrifuged at 450 g for 10 min, and the lower phase was transferred to petri dish and put in the acid fume hood for 1 h or until there was no chloroform left. The extracted oils were transferred to 1.5 ml vial and centrifuged at 8000 xg for 1 minute and the supernatant was stored in new vials for further use. Samples were diluted to 12.5–500 µg/mL in concentration.

### Gas chromatography (GC) analysis

The analysis was done by PT Saraswanti Indo Genetech to identify polyunsaturated fatty acid (PUFA) in tempeh oil. The tempeh oil is prepared with adding 1.5 mL 0.5 M KOH in falcon tube and vortexed. The mixture was heated at 100 ℃ for 20 min. After the mixture in room temperature, 1.5 mL of 20% BF_3_ in methanol was added and vortexed. The mixture was heated back at 100 ℃ for 20 min. The mixture was cooled down to 30 ℃, and 3 mL saturated NaCl and 2 mL hexane were added and then vortexed for 2 min. There were 2 layers formed, and the 2 mL of upper layer was transferred to new tube that consisted of Na_2_SO_4_ anhydrous and incubated for 15 min. The 0.1 µL mixture was injected to GC system with injection temperature 24 ℃, DB FastFAME capillary column, helium gas, gradient temperature 50–230℃, running time 24.67 min, FID 240 ℃ detector, helium gas flow rate 30 mL/minute, and air flow rate 300 mL/min.

### Antioxidant activity

Analysis antioxidant of tempeh oil was conducted by ferric reducing antioxidant power (FRAP) method according to Sharopov et al. [27] using ferric salt tripyridyltriazine (TPTZ) as the oxydator. The FRAP reagent was freshly prepared with 300 mM acetic buffer pH 3.6, 10 mM TPTZ (in 40 mM HCl), 20 mM FeCl_3_ (in aquadest) with ratio of 10:1:1. The FRAP reagent was incubated at 37 ℃ for 30 min, followed by mixing 20 µL of sample dissolved in DMSO and 180 µL FRAP reagent, and incubated for 6 min. Mixture of FeSO_4_ (9.375–1200 µM) was used to obtain the standard curve. The absorbance was read using microplate reader at 595 nm wavelength. The assay was performed in triplicate.

### Anticholinesterase activity

Inhibition of cholinesterase activity was conducted by Ellman’s assay (modified method of Ovais et al. [28]). Acetylcholinesterase (AChE) and butyrylcholinesterase (BChE) activities were tested with 5 samples (galantamine, donepezil, tempeh oil, soybean oil, and omega-3 supplement) in various concentrations (12.5–500 µg/mL diluted with 70% ethanol). Powdered AChE and BChE enzyme were diluted with 50 mM tris-HCl pH 8 (buffer A) to reach the final concentration of 0.22 U/mL. The assay consisted of 25 µL sample, 125 µL 0.3 mM 5,5′-dithiobis [2-nitrobenzoic acid] (DTNB) in 50 mM tris-HCl pH 8 with addition 0.1% bovine serum albumin (BSA) (buffer B), 50 µL 50 mM tris-HCl pH 8 with addition of 0.1 M NaCl and 0.02 M MgCl_2_.6H_2_O (buffer C), and 25 µL 5 mM acetylthiocholine iodide (ATCI) in aquadest. The mixture was then incubated at 37 ℃ for 10 min. The AChE or BChE enzyme was added for 25 µL, and the absorbance was read immediately at 412 nm wavelength in 11 cycles with 30 sec interval. The assay was performed in triplicate.

$${\text{Anticholinesterase}}\;{\text{activity }}\left( \% \right) \, = \frac{{{\text{negative}}\;{\text{controls}}{ - }{\text{sample}}}}{{{\text{negative}}\;{\text{control}}}} \times 100\%$$

### Cell preparation

The cells preparation was done with Zhai et al. [29] as reference. Dulbecco’s modified eagle medium (DMEM) which supplemented with 10% fetal bovine serum (FBS) and 1% streptomycin/penicillin was used. The cryopreserved cell vial was thawed quickly with 37 ℃ water bath. The cells were transferred to 15 mL tube which contained 5 mL of growing media and then centrifuged at 1000 g for 3 min. The supernatant was removed, and 5 mL growing media was added, and cells were cultured to the flask. Cells were incubated at 37 ℃ and 5% CO_2._ Media was changed every 3 days. After cell confluent reached 95%, the cells were subcultured to new flask.

### Cell viability assay and cell treatment

The assay was used to evaluate the effect of samples and lipopolysaccharide (LPS) that was treated to the cells. This experiment was performed according to Guo et al. [30] with modification. The cells were seeded to 96-well plate at seeding density 1.0 × 10^4^ cells/well and incubated for 24 h. The media was changed to DMEM free serum media to reduce the mitogenic effect. The samples were diluted with 1% DMSO to final concentrations of 50 and 100 µg/mL and were added to the cell for 1 h, followed by adding the LPS at final concentration of 1 µg/mL. The negative control was cell without sample and LPS and incubated for 24 h. The positive control was cell treated with LPS only. Next, the media was discarded and 20 µL of 5 mg/mL 3-(4,5-dimethylthiazol-2-yl)-2,5-diphenyltetra-zolium (MTT) was added. The cells were incubated for 3 h, and 100 µL DMSO was added. The absorbance was read at 560 nm wavelength using microplate reader.

The cell treatment procedure was done in 24-well plate to get more cells yield for gene expression analysis. The cells at seeding density 1.0 x 10^5^ cells/well were seeded. After grown for 2 days or 80% confluent, the cells were given with samples (4 samples, negative control, positive control) for 1 h and followed by adding the LPS with final concentration 1 µg/mL. The cells were incubated for 24 h harvested and stored in 1.5 mL vial in freezer for further analysis.

### Gene expression analysis with quantitative PCR (qPCR)

The experiment was performed according to Fourrier *et al.* (2017) with modification [31]. The cells that have been treated were lysed using GENEzol Reagent and RNA was extracted. The RNA was then used as template for cDNA synthesis. Two-step RT-PCR was done in this experiment. The cDNAs were analyzed with specific primer genes related to Alzheimer’s disease, including *TNF, cdk5, gsk3b, PSEN1, PSEN2,* and *β-actin* as internal control (Table 1). The primer sequences were designed with primer and designing tool which provided by NCBI. The primers were run an optimization procedure with PCR electrophoresis. Quantification analysis used qPCR and master mix kit. The reaction was 20 µL in volume with 40 cycles of 92 ℃ for 5 sec 55 and 58 ℃ for 30 sec, 72 ℃ for 30 sec. The relative quantification (RQ) was calculated with the comparative CT method.

**Table 1:** Specific primer sequences

No	Gene	Annealing temperature (°C)	Amplicon (bp)	Sequence
1	*TNFα*	55	319	F: 5'GGCAGGTCTACTTTGGAGTCATTG'3
				R: 5'ACATTCGAGGCTCCAGTGAATTCGG'3
2	*cdk5*	55	360	F: 5’ ACTGTGTTCAAGGCTAAAAACC ‘3
				R: 5’ CAATTTCAACTCCCCATTCC ‘3
3	*Gsk3b*	58	368	F: 5'CGAACTCCACCAGAGGCAAT'3
				R: 5'TAAGTGCTGGTCTTCCCTGC'3
4	*PSEN1*	58	485	F: 5' TAATGGCCGACCCCAGAGTA'3
				R: 5'CAATCATCCCGACCACACCA'3
5	*PSEN2*	58	314	F: 5' GTCTGATGAGCTGAGAGCCAG'3
				R: 5'TGCTTCGCCCCATACTTGAG'3
6	*B-actin*	58	439	F: 5'TGGAATCCTGTGGCATCCATGAAAC'3
				R: 5'TAAAACGCAGCTCAGTAACAGTCCG'3

$${\text{RQ }} = \, \left( {{2}^{{ - \Delta \Delta {\text{CT}}}} } \right)$$

### Statistical analysis

The experiments were carried out in triplicate, and all data that have been collected were analyzed statistically using SPSS application. The data are represented as mean ± standard deviation (SD). The significance of differences is analyzed by one-way analysis of variance (ANOVA) with *p* value less than 0.05.

## Result and discussion

### Yield of Tempeh oil and PUFA identification

**Fig. 2:**
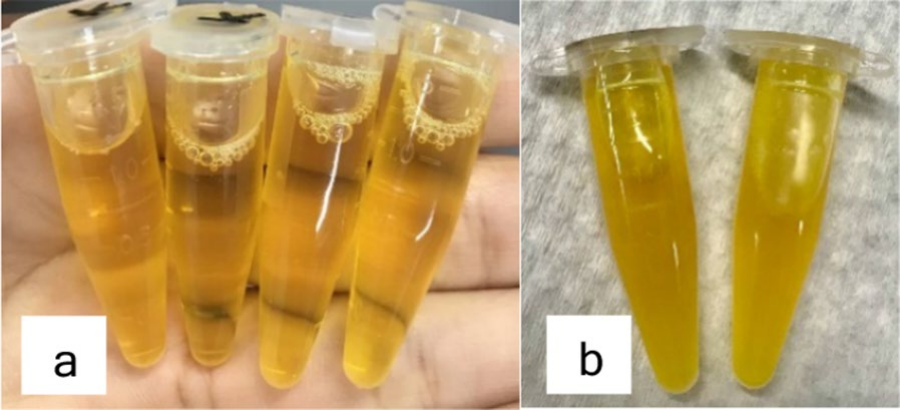
Soybean oil extract (**a**); tempeh oil extract (**b**)

The yield of soybean and tempeh oil (Fig. 2) was calculated as ratio of total weight of oil to the soybean/tempeh powder after collected from the freeze dryer. The percentage of crude soybean and tempeh oil extract yield were 7.14% (w/v) and 70% (w/v). However, tempeh oil was extracted with 96% ethanol maceration method and rotary evaporator. The major component of PUFA in the oils was dominantly represented by omega-6. However, 8.37% omega-3 component was identified in tempeh oil which has not found from the previous study by Subali et al. [21] (Table 2).

**Table 2:** Major PUFA in soybean and tempeh oil

Parameter	Result	
	Soybean oil (%)	Tempeh oil (%)
α-Linolenic acid (Omega-3)	8.20	8.37
Linoleic acid (Omega-6)	54.77	53.14

The method of extraction process suggested by Subali et al. [21] was effective. The powdered tempeh had more surface area which gave easier contact access for the solvent. However, the total yield could not be as much as 24 h maceration technique that was applied in the middle of the experiment. In Bligh-Dyer method, there were three layers formed, including polar phase, interphase contained unextractable sample residue, and nonpolar phase contained lipid. Some of the lipids were trapped in the interphase, and it was hard to get all of it to the lipid layer. For the PUFA compound identification, α-linolenic acid that was belonged to the omega-3 family was identified in tempeh oil for 8.37%. α-linolenic acid (ALA) predominantly found in plants is a precursor compound to eicosapentaenoic acid (EPA) and docosahexanoic acid (DHA) which mainly present in marine. ALA can be converted to EPA and DHA by a desaturase enzyme [32]. However, according to Brenna et al. [33], the conversion efficiency of ALA to EPA and DHA was varied from 0 to 21% between individuals. The quantity of ALA in tempeh oil almost like the soybean oil did not go through the fermentation process. This was proved that the tempeh culture did not consume the omega-3 during the fermentation process.

Thus, ALA can be suggested to have minor role in reducing Alzheimer’s disease risk [34]. A study by Handajani et al. [35] also suggested that consuming tempeh could improve mild cognitive impairment in elderly.

### Anticholinesterase activity of tempeh oil

For the acetylcholinesterase (AChE) inhibition test, all the samples showed inhibitory effect in all concentration except for omega-3. The most AChE inhibition activity of galantamine and donepezil was 77.63% and 78.32%, both from 500 ug/mL concentration. However, the highest AChE inhibition of soybean oil was 33.15% in 250 ug/mL and tempeh oil was 33.58% in 25.0 ug/mL (Table 3). Through statistical analysis, all concentration in tempeh oil was significantly different to all soybean oil concentration.

**Table 3:** Anti-acetylcholinesterase activity of tempeh oil

Sample	Anti-acetylcholinesterase activity (%)					
	12.5 µg/ml	25 µg/ml	50 µg/ml	100 µg/ml	250 µg/ml	500 µg/ml
G	65.69 ± 2.83	68.23 ± 1.25	73.73 ± 0.62	77.00 ± 2.00	77.03 ± 0.87	77.63 ± 0.34
D	51.89 ± 2.88	62.27 ± 1.12	71.15 ± 1.12	71.85 ± 1.32	76.26 ± 0.65	78.32 ± 1.64
O	–	–	–	–	–	20.42 ± 1.37
SO	26.84 ± 1.07^b,c^	31.60 ± 8.57^c,d,e^	28.57 ± 0.12*	28.67 ± 1.71*	33.15 ± 5.25^d,e^	28.89 ± 1.23*
TO	29.62 ± 1.76*	33.58 ± 3.65^e^	27.26 ± 2.29^b,c,d^	28.84 ± 4.90*	30.69 ± 2.82*	24.71 ± 2.31^a,b^

“- ” no inhibition activity, * = ^b,c,d,e^, G = galantamine, D = donepezil, O = omega-3, SO = soybean oil, TO = tempeh oil

For the BChE inhibition test, all the samples showed inhibition potential toward the BChE enzyme but not as high as AChE inhibition. The highest inhibition in galantamine was 52.34% in 250 ug/mL, and donepezil was 39.25% in 500 ug/mL. Meanwhile, soybean oil had 19.72% and tempeh oil had 26.67% inhibition both in 500 ug/mL (Table 4). Acetylcholine is a neurotransmitter in the human brain that has an important role for signaling transfer between synapses. Acetylcholine also has a function to maintain a person’s memory, behavior, and cognitive. The presence of AChE and BChE can make the ACh level to decrease and lead to elevation of Alzheimer’s risks [28]. In present, one of the treatments related to neurological disorders is to inhibit the activity of cholinesterase enzymes. Several drugs such as galantamine and donepezil have been used to treat Alzheimer’s focusing on inhibition of cholinesterase. The available drugs have many side effects, and some can cause hepatotoxicity.

**Table 4:** Anti-butyrylcholinesterase activity of tempeh oil

Sample	Anti-butyrylcholinesterase activity (%)					
	12.5 µg/ml	25 µg/ml	50 µg/ml	100 µg/ml	250 µg/ml	500 µg/ml
Galantamine	15.99 ± 1.84	23.33 ± 4.81	36.47 ± 5.37	44.66 ± 1.28	52.34 ± 3.05	51.18 ± 0.30
Donepezil	–	–	3.54 ± 0.79	5.40 ± 1.19	22.40 ± 2.77	39.25 ± 5.15
Omega-3	29.14 ± 0.53	13.10 ± 2.31	12.02 ± 3.24	8.26 ± 4.89	–	7.65 ± 0.86
Soybean Oil	19.04 ± 4.28	18.05 ± 2.06	9.25 ± 1.40	19.30 ± 7.28	17.53 ± 2.37	19.72 ± 2.43
Tempeh Oil	23.30 ± 3.26	21.92 ± 0.56	17.85 ± 9.54	22.13 ± 5.39	25.78 ± 1.20	26.67 ± 2.60

Not only the side effects are being concerned but also its effectiveness does not work in mild Alzheimer’s [25, 36]. In the current study, tempeh oil 25 µg/mL was demonstrated to have the most inhibition activity toward acetylcholinesterase and was not significantly different to soybean oil in same concentration. Compounds that were dissolved in tempeh oil extract including PUFAs and aglycones could be played a role in the cholinesterase inhibition. It had not been determined which compound majorly contributed to the inhibition. The previous study by Ahmad *et al.* (2015) reported that aglycones in tempeh extracted with *n*-hexane were higher than the soybean extract [37].

Butyrylcholinesterase (BChE) is a hydrolase enzyme in plasma which can hydrolase the acetylcholine (ACh) neurotransmitter well but not as effective as AChE. BChE also could degrade bioactive esters compound that mainly is found in plants. Some research had reported that BChE has another role aside from degrading ACh. BChE has the capability to hydrolase ghrelin which is a hormone that is responsible in hunger and hydrolase cocaine. The reports showed that lack of BChE function was found in people with obesity, cocaine addiction, anxiety, and aggression [38]. The current study was demonstrated that the inhibition power of galantamine and donepezil in BChE was not as powerful as it was in AChE. The main factors affecting the inhibition power were the drug’s target. According to the drug description in the box, galantamine and donepezil are used specifically to inhibit the AChE activity. Hence, it was corresponding to the aim of the study that the tempeh oil had the ability to inhibit the AChE but not as powerful as in BChE, since the human body needs the BChE enzyme to degrade ghrelin and cocaine.

### Antioxidant activity of tempeh oil

**Fig. 3:**
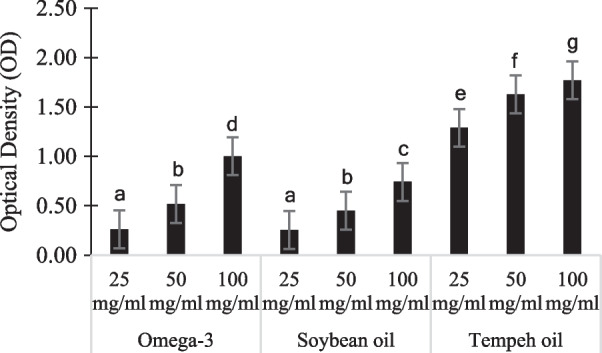
Antioxidant activity from tempeh oil compared to commercially omega-3 and soybean oil extract

The antioxidant activity test was done with ferric reducing antioxidant power (FRAP). In FRAP assay, the antioxidant level in samples was evaluated through the ability of samples to donate one electron. The oxidation number of Fe^3+^ in FRAP reagent would be reduced to Fe^2+^ and thus change the assay color to dark purple [27]. The experiment showed that tempeh oil had the most antioxidant activity compared to omega-3 and soybean oil in all concentrations and was significantly different (Fig. 3).

In Alzheimer’s disease, oxidative stress is one of the factors that participates in the early stage of the disease. Not only that, but the oxidative stress also plays a role in activating several signaling pathways to cause cell damage and promote the progress of Alzheimer’s disease. Consuming food that has antioxidant becomes a diet to lower the risk of developing Alzheimer’s. Many research had been focusing to use antioxidant as therapies for Alzheimer’s [39]. According to Hashim *et al.* (2018), wrapping the soybean during the fermentation process could elevate the antioxidant level in tempeh product [40]. This experiment had shown that tempeh oil had the highest ability to scavenging free radicals compared to commercial omega-3 tablet that would be inhibited the oxidative stress to develop. The commercial omega-3 supplement had been purchased from DAISO, and the ingredients not only have omega-3 extract but also other compounds such as vitamin B12, maltodextrin, protein, crystalline cellulose, and calcium stearate. Thus, commercial omega-3 had lower antioxidant levels than tempeh oil extract.

### Viability cell on treated neuronal Schwann cells with tempeh oil and LPS

**Fig. 4:**
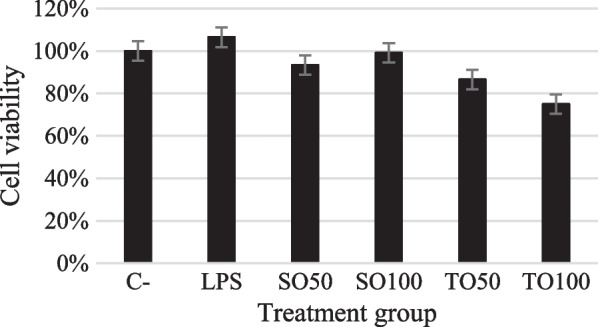
Cell viability of soybean oil (SO50, SO100), tempeh oil (TO50, TO100), and LPS

MTT assay was used to evaluate the effect of tempeh oil and LPS to the viability of RSC96 cells (Fig. 4). Data showed there was no extreme cytotoxicity from soybean oil, tempeh oil, and LPS treatment. The cells were exposed to the samples first followed by the LPS to see how the samples would reduce the LPS-induced inflammation. Based on Guo et al. [30], LPS-induced inflammation could elevate the TNF-a, IL-6, and IL-1B cytokine [30].

### Effect of tempeh oil extract on gene expression related to Alzheimer’s disease in LPS-induced Schwann cells

**Fig. 5:**
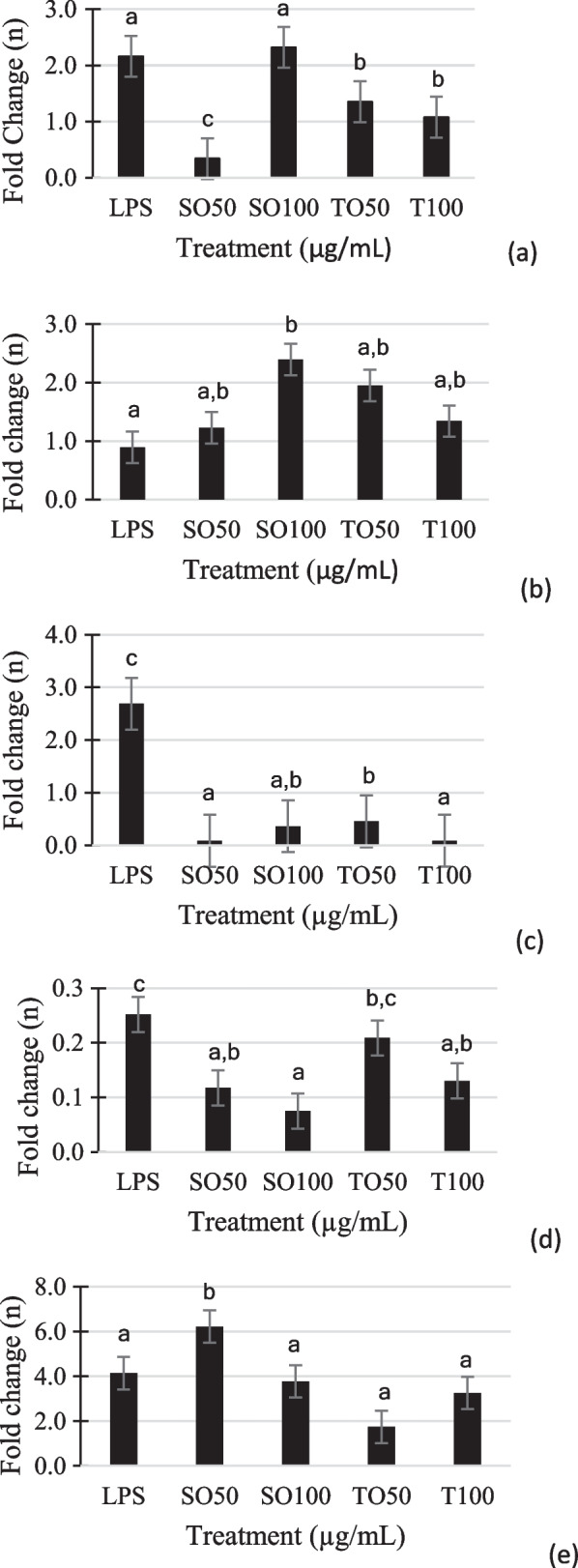
Tempeh oil effect on gene expression in LPS-induced Schwann cells: **a** *PSEN1*, **b** *PSEN2*, **c** *Gsk3b*, **d** *cdk5*, (e)*TNF*

The expression of genes related to Alzheimer’s disease was quantified using qRT-PCR (Fig. 5). LPS-induced neuronal Schwann cells were used to mimic the Alzheimer’s disease. The quantification showed that tempeh oil (TO50, TO100) had the ability to downregulate the expression of *PSEN1, Gsk3b,* and *cdk5* compared to the positive control. The concentration of tempeh oil also effected the inhibition activity which came out higher in 100 µg/mL concentration.

This study was the first experiment in examining the difference effects on the expression of gene related to Alzheimer’s with LPS-induced cells that had been treated with soybean oil extract and tempeh oil extract *in vitro*. The *PSEN1* (presenilin-1) and *PSEN2* (presenilin-2) gene encodes protein for γ-secretase enzyme. The enzyme plays a role in producing the amyloid-β protein which can aggregate in the brain and forming a plaque that will interrupt signaling process between synapses. The amyloid-β protein is produced from protein called APP (amyloid precursor protein). Excessive production of amyloid-β protein can lead the individual to develop the pathogenic pathways of Alzheimer’s [2]. In *PSEN1* mRNA, tempeh oil at concentration of 100µg/mL could downregulate up to 2 times compared to the control. However, note there was no statistically significant difference in any *PSEN1* gene expression data. Soybean oil at 50 µg/mL had more ability to suppress the PSEN1 mRNA, but with concentration of 100 µg/mL the expression was upregulated. Although the soybean oil had stronger inhibitor ability, rising the treatment dosage could upregulate the expression. Meanwhile in tempeh oil, rising the dosage could further downregulate the *PSEN1* mRNA. For the *PSEN2* mRNA, no sample could downregulate the mRNA compared to the control. The expression of all samples was upregulated. Cells treated with soybean oil at concentration of 100 µg/mL had the highest mRNA expression. The same case in *PSEN1* mRNA happened with *PSEN2* mRNA, in which a higher dose of soybean oil enhanced the expression, but conversely happened with tempeh oil. In this preliminary study, a concentration of 100 µg/mL seemed to show limited suppression of *PSEN1* gene expression. Although it is necessary to study in more depth the mechanism of the active compounds in soybean oil to inhibit *PSEN1*, it seems that the active compounds at 100 ug/mL were too high and less to be effective in inhibiting *PSEN1*. According to Delabio et al. [13] that had been evaluated the mRNA of *PSEN1* and *PSEN2* in parts of the brain, the postmortem study reported patients with Alzheimer’s had more expression in PSEN1 and low expression in PSEN2 compared with healthy elderly patient [13]. The study of Li et al. [41] reported that an increase in *PSEN1* expression was sufficient to elevate the activity of γ-secretase and thus worked up the production of amyloid-β protein. Also, the activity of γ-secretase is specifically higher in PSEN1-containing enzyme than PSEN2-containing complex [41].

The other cause of Alzheimer’s disease is including the hyperphosphorylation of tau protein. Normally, human body produces the tau protein and tau phosphorylation usually happens. However, in Alzheimer’s the tau protein becomes hyperphosphorylated and the kinase enzymes responsible for the event to happen are glycogen synthase kinase 3 beta (Gsk3b) and cyclin-dependent kinase 5 (cdk5) [42]. Based on Das et al. [43], gsk3b enzyme was the key of tau hyperphosphorylation. The study proved that in AD rat brains, the tau phosphorylation was increased significantly causing the neurofibrillary tangles accumulation [43]. In this study, the tempeh oil could significantly suppress the expression of *Gsk3b* mRNA. Higher dosage of tempeh oil 100 µg/mL had downregulated the expression up to 29 times compared to the control. Although soybean oil 50 µg/mL had a similar ability to downregulate the expression, in higher dosage the ability was reduced. All the samples could strongly inhibit the *Gsk3b* gene expression. According to Niculescu *et al.* (2020), healthy individuals had lower number of Gsk3b expression and higher expression in people with memory disorders [44]. Meanwhile, the activity of cdk5 enzyme is activated by protein activators including p35, p39, p29, p25, and p10. To be noted, p35 can be cleaved to p25 and p10 in which the p25 will lead the cdk5 activity that becomes stable and uncontrolled. The cdk5 that has been activated by p25 will be actively phosphorylated the tau protein generating the neurofibrillary tangles. Cleavage of p35 can happen when p35 is exposed to neurotoxicity conditions [45, 46]. Therefore, minimizing the cdk5 activity or p25 level in brains can reduce the risk of Alzheimer’s disease. In this study, all samples could downregulate the *cdk5* gene expression with the highest inhibition in soybean at 100 µg/mL. Although soybean oil had the inhibition ability, tempeh oil could be claimed to have the capability to suppress the transcription of *cdk5* gene with considering the health benefits that had been explained previously.

Much research had been reported that cytokines were associated with Alzheimer’s pathogenesis including tumor necrosis factor a (TNF-α). The TNF-α is classified as pro-inflammatory cytokine. Researchers have found that TNF-α accumulated around the amyloid-β plaques and alleviated in patients with Alzheimer’s. The TNF-α cytokine was likely to more affect the amyloid-β protein level than phosphorylation of tau protein level [47]. The current study was demonstrated that tempeh oil at 50 µg/mL had the potential to suppress the LPS-induced TNF-α gene expression up to 2.7 times and was significantly different compared to control in Schwann cells. Tempeh oil at 100 µg/mL could inhibit the gene expression up to 1.3 times compared to control. Both concentrations of tempeh oil had the ability to downregulate the pro-inflammatory cytokine gene expression. Correlation between treatment dosages need to be evaluated further. According to Nakajima et al. [48], white soybean tempeh had 15.7 mg of total aglycones (daidzein, glycitein, genistein) per 100 g of wet weight. In theory, aglycone is a water-insoluble compound which needs to be extracted with organic solvents, such as ethanol and chloroform. The aglycone compounds have been proven to possess anti-inflammatory activity, thus explaining how the tempeh oil could minimize the gene expression [49]. Meanwhile, many studies have reported the long chain of omega-3 fatty acids also proved to have the activity of reducing inflammation in the early stage of Alzheimer’s disease [34].

### Tempeh: The superfood and its connection to the past

Historically, tempeh was originated from ancient Java. As recorded by Dutch botanist, Rumphius (1747 A.D.), the Chinese traders introduced soybeans to the Javanese society, and since then, they began to incorporate soybeans as foods and fertilizer [15]. The word “*tempe*” was first found in the classic Javanese literature, Serat Centhini, vol. 3 (1814), where tempeh was served as a royal food in ancient Javanese royal family of Sunan Giri in Central Java, Indonesia [50]. The word “*tempe*” was also found in the Javanese-Dutch dictionary, described as the side dish made from soybean or press cake (bungkil) fermentation [51].

In the early nineteenth century, Javanese society experienced a rapid increase in population and land became scarce [52]. Since the late of 1600s, Indonesia had been colonized by the Dutch and up to 80% of the Javanese population was forced to work in crop plantation areas. The society experienced food scarcity during this time, and the quality of their diets decreased. Later, people relied on tempeh as a source of nutrition to survive the forced labor camps [51, 52]. Since then, tempeh has become an integral part of the Javanese cuisine and later spread throughout the nations. Tempeh has long been hailed as superfood due to its high nutritional values, including crude and soluble proteins (up to 16% and 66%, respectively), fat, crude fibers, micronutrients, and antioxidants and has been proven to prevent and combat various diseases [18].

### The post-pandemic establishment of tempeh in the food industry

Food security has been a challenge to the world as global population increase. Food productions are ensured to be accessible, sustainable, safe, healthy, and equitable for everyone, primarily focusing on reducing hunger and malnutrition to achieve a greater health level of the population [53]. In FAO’s The State of Food Security and Nutrition in the World 2022, agrifood systems are expected to be transformed and become more resilient in delivering sustainable and inclusive low-cost nutritious foods for the nations [54].

After two years of the COVID-19 pandemic, the world is still facing various challenges in the food security systems, as the economic crisis arose worldwide, triggering global food crisis [53]. Considering sustainability and the environmental impact of the animal agriculture, as the demand for better quality foods is increasing, it cannot be met by animal protein alone, but also plant-derived ones [55]. Tempeh has been considered as an affordable, nutritious, and sustainable protein source [56]. Tempeh makes a perfect meat alternative.

Indonesia is the world’s largest tempeh producer and Asia’s largest soybean market. Tempeh makes up to 50% of Indonesia’s soybean consumption. In Indonesia alone, averagely people consume up to 6.45 kg tempeh per person every year [57]. Tempeh has also gained popularity in other countries due to its high nutritional values. Valued at US$3.7 billion in 2020, the global tempeh market is projected to reach a revised size of US$5.3 billion by 2027 amid the COVID-19 crisis [58]. The COVID-19 pandemic has accelerated market focusing on food bioactives, supplements, and nutraceuticals in supporting human immune systems, since there has been no precise cure developed for COVID-19 [59]. Macronutrients, like carbohydrates, proteins, and fats, can directly modulate the immune cells to combat viral infections. Omega-3 fatty acids, if taken adequately, may protect our cells against bacterial and viral infection through the modulation of immune responses (including CD8+ T cells) [60]. This study, to some extent, has analyzed the PUFAs content, as well as the antioxidant activities of tempeh and soybean oil. It is hoped that this study can enrich the knowledge about nutritional benefits of tempeh, mainly focused on combating Alzheimer’s disease, and modulating immune systems for overall well-being. Along with increasing research on tempeh, as the awareness of people increases, tempeh can be viewed not only as a portion of cultural heritage originating from Indonesia but also as an affordable, highly nutritious food that may be one of the solutions to the food sustainability challenges during COVID-19 pandemic and beyond.

## Conclusion

This study suggested that tempeh oil that was extracted from white soybean tempeh had PUFA compounds including omega-3 and omega-6 and had the ability to significantly downregulate the Alzheimer’s disease gene-related expression that were *PSEN1, Gsk3b, cdk5,* and *TNF*. From the findings, tempeh oil may have the potential of lowering the risk of individuals to develop Alzheimer’s disease. Hence, tempeh oil was better than soybean oil in reducing the gene expression and had more antioxidant activity. However, further study needs to be done obtain tempeh oil whole bioactive compounds, evaluate orally treated Alzheimer’s disease model, and correlation between treatment dosages.

## Data Availability

Not applicable.
